# A multidisciplinary approach to prevent and treat pressure sores in proned COVID-19 patients at a quaternary university hospital

**DOI:** 10.6061/clinics/2020/e2196

**Published:** 2020-08-06

**Authors:** Fabio de Freitas Busnardo, Gustavo Gomes Monteiro, Rogério Rafael da Silva Mendes, Laielly Abbas, Vitor Figueiredo Pagotto, Cristina Camargo, Maria José Carvalho Carmona, Rolf Gemperli

**Affiliations:** Divisao de Cirurgia Plastica, Faculdade de Medicina FMUSP, Universidade de Sao Paulo, Sao Paulo, SP, BR.

An estimated 10-15% of patients with respiratory disease resulting from coronavirus disease 2019 (COVID-19) develop acute respiratory failure and require intensive care (1). In order to improve breathing patterns in patients with severe acute respiratory syndrome (SARS), prone positioning can make ventilation more efficient. Proning minimizes ventral alveolar distension and reduces dorsal alveolar collapse by decreasing the difference between dorsal and ventral transpulmonary pressure. It also prevents compression of the lungs, consequently improving blood perfusion and gas exchange (2).

When indicated, proning should begin early (preferably within the first 24 hours) in patients who present with SARS and serious deterioration of gas exchange characterized as a ratio of the partial pressure of oxygen in arterial blood to the fraction of inspired oxygen (PaO2/FiO2) below 150 mmHg. Once patients are placed in the prone position, they must remain prone for at least 16 hours (up to 20 hours) before being returned to the supine position (3).

However, a serious potential complication of proning is pressure lesions, which develop in anatomical regions different from those typically seen in bedridden patients. Involvement in the malar, nasal, and frontal regions as well as the jaw, lips, sternal region, and iliac crests is not uncommon as these hemodynamically labile and unstable patients remain in a fixed position for up to 20 hours per day as a life-saving measure. Additional risks include facial edema, transient hemodynamic instability, corneal abrasions, and obstruction of the endotracheal tube (4).

As the pandemic was already well-established with an increased number of cases in March 2020, the Central Institute at the Hospital das Clínicas in the University of São Paulo School of Medicine, the largest and oldest institute in Latin America’s largest hospital complex, began to exclusively treat COVID-19 patients with 900 inpatient beds reserved for this group. Initially, this effort involved 200 intensive care beds, which later expanded to 400. In this unprecedented situation, the plastic surgery service at the same hospital began to draw attention as numerous complications emerged due to the positioning of these patients, and the long periods they were confined to their beds.

Intensivists observed an improvement in the ventilatory parameters of proned patients, but the cost was a high incidence of pressure lesions, which were initially seen in up to 80% of patients who were placed in the prone position for more than 16 hours per day. In response, the plastic surgery group formed a multidisciplinary team to study, prevent, and treat pressure lesions in proned patients. This group included professionals from anesthesiology, pulmonology, nursing, physiotherapy, and the Hospital das Clínicas technical innovation team (CITIC-InovaHC, coordinated by the plastic surgery department).

First, written material was produced to provide information about this topic to the hospital’s intensive care unit staff. Then, a special team was created for daily evaluation and treatment of severe cases. This team comprised 11 physicians (10 resident physicians in plastic surgery, directed by one assistant surgeon), a physiotherapist, and a wound care nurse. In addition to the follow-up care of these patients, this team was also responsible for surgical treatment when necessary.

Measures to prevent pressure lesions involve a multidisciplinary approach and require daily inspection, hygiene, hydration, control of humidity and skin temperature, and most importantly, reducing pressure on bony prominences. This requires a change in body position every 2 hours, which is often impossible when patients are in a serious and unstable condition. In response, protective pads were developed specifically for each case. When adapted correctly and made in partnership with the private initiative, they proved to be the best alternative for patients kept in the prone position for long periods.

Providing extra care for proned patients can help them overcome the complex changes in pulmonary physiology. Within the context of plastic surgery at a quaternary care hospital, preventing pressure lesions in these patients is extremely important since this complication is directly related to reduced quality of life, longer hospital stays, increased public and private spending on health, and lower indicators related to the quality of care provided.
